# Neural Progenitor Cell Implants Modulate Vascular Endothelial Growth Factor and Brain-Derived Neurotrophic Factor Expression in Rat Axotomized Neurons

**DOI:** 10.1371/journal.pone.0054519

**Published:** 2013-01-18

**Authors:** Rocío Talaverón, Esperanza R. Matarredona, Rosa R. de la Cruz, Angel M. Pastor

**Affiliations:** Laboratorio de Fisiología y Plasticidad Neuronal, Departamento de Fisiología, Facultad de Biología, Universidad de Sevilla, Sevilla, Spain; Florey Institute of Neuroscience & Mental Health, Australia

## Abstract

Axotomy of central neurons leads to functional and structural alterations which largely revert when neural progenitor cells (NPCs) are implanted in the lesion site. The new microenvironment created by NPCs in the host tissue might modulate in the damaged neurons the expression of a high variety of molecules with relevant roles in the repair mechanisms, including neurotrophic factors. In the present work, we aimed to analyze changes in neurotrophic factor expression in axotomized neurons induced by NPC implants. For this purpose, we performed immunofluorescence followed by confocal microscopy analysis for the detection of vascular endothelial growth factor (VEGF), brain-derived neurotrophic factor (BDNF), neurotrophin-3 (NT-3) and nerve growth factor (NGF) on brainstem sections from rats with axotomy of abducens internuclear neurons that received NPC implants (implanted group) or vehicle injections (axotomized group) in the lesion site. Control abducens internuclear neurons were strongly immunoreactive to VEGF and BDNF but showed a weak staining for NT-3 and NGF. Comparisons between groups revealed that lesioned neurons from animals that received NPC implants showed a significant increase in VEGF content with respect to animals receiving vehicle injections. However, the immunoreactivity for BDNF, which was increased in the axotomized group as compared to control, was not modified in the implanted group. The modifications induced by NPC implants on VEGF and BDNF content were specific for the population of axotomized abducens internuclear neurons since the neighboring abducens motoneurons were not affected. Similar levels of NT-3 and NGF immunolabeling were obtained in injured neurons from axotomized and implanted animals. Among all the analyzed neurotrophic factors, only VEGF was expressed by the implanted cells in the lesion site. Our results point to a role of NPC implants in the modulation of neurotrophic factor expression by lesioned central neurons, which might contribute to the restorative effects of these implants.

## Introduction

Axotomy of adult CNS neurons leads to severe morphofunctional alterations which include synaptic stripping and reduced firing rate [1], [2]. When neurotrophic factors are delivered to the distal stump of lesioned nerves, these alterations are largely prevented and neuronal function is restored [3], [4], [5], [6], which suggests that physiological changes induced by axotomy are, at least in part, due to the lack of target-derived trophic support. Neurotrophic dependence of adult neurons is also evidenced by the axotomy-induced regulation in the expression of growth factors and/or their receptors [7], [8], [9], which might compensate for the loss of retrograde trophic support through alternative pathways, such as anterograde, paracrine or autocrine [10].

By using the oculomotor system as the experimental model, we have previously described that the transection of the medial longitudinal fascicle (MLF) induces profound alterations in the axotomized internuclear neurons of the abducens nucleus, which are left disconnected from their target [2], [11]. The abducens nucleus is composed of motoneurons that innervate the ipsilateral lateral rectus muscle, and a group of premotor cells, the abducens internuclear neurons, whose axons course through the MLF to establish synaptic contacts with medial rectus motoneurons in the contralateral oculomotor nucleus [12]. After their axotomy, abducens internuclear neurons show a marked reduction in firing rate, a significant decrease in eye position and velocity sensitivities and a noticeable stripping of synaptic afferences [2], [11]. Moreover, when a new target (embryonic tissue) is provided, lesioned abducens internuclear neurons are able to restore their firing properties and synaptic afferences [13], [14]. It was suggested that neurotrophic factors released by the implanted tissue probably mediate the re-establishment of these axotomy-induced alterations, a hypothesis supported by the fact that abducens internuclear neurons express the different types of trk receptors for neurotrophins [15].

As a further step, we have explored in our central axotomy model the effects of neural progenitor cell (NPC) implants based on the reported efficacy of this cell population in promoting functional improvement of damaged CNS neurons [16], [17], [18]. Thus, axotomized abducens internuclear neurons largely recover their morphophysiological properties following the implant of NPCs at the lesion site in the MLF, as we have shown recently in the cat [19]. Several mechanisms have been proposed to be likely involved in the benefitial effects of the NPC implants after CNS injury: secretion of neurotrophic and/or angiogenic factors, restoration of synaptic transmitter release by providing local reinnervation, re-establishment of functional afferent and efferent connections, or change in the brain microenvironment towards more favourable conditions for both the lesioned neurons to survive and the implanted progenitors to integrate [20], [21]. In line with this, recent evidence suggests that implanted NPCs establish a “cross-talk” or bilateral communication with host tissue cells of relevant importance for the neuroprotective action of the implants [22], [23].

NPCs used in our study were obtained from the subventricular zone (SVZ) of postnatal animals. In their neurogenic niche, SVZ cells have been described to express the angiogenic factor vascular endothelial growth factor (VEGF) and the neurotrophic factors brain-derived neurotrophic factor (BDNF), nerve growth factor (NGF) and neurotrophin-3 (NT-3) [24]. More precisely, SVZ astrocytes express all the mentioned angionenic and neurotrophic factors whereas SVZ neural progenitors selectively express VEGF [24]. The neurobiological interest for VEGF is sharply increasing nowadays since in addition to its well known angiogenic activity, VEGF also exhibits neurotrophic and neuroprotective properties [25], [26], [27], [28]. Moreover, a role in synaptic transmission has also been attributed to this factor. In particular, VEGF is able to induce a depression in excitatory synaptic transmission onto pyramidal and granule neurons of the hippocampus and also on hypoglossal motoneurons [29], [30]. On the other hand, the neurotrophins BDNF, NT-3 and NGF, also expressed in the SVZ niche, have been demonstrated to induce benefitial effects in lesioned neurons after different types of brain damage [3], [5], [6].

In the present work, we aimed to investigate possible modifications of neurotrophic factor expression in central injured neurons following NPC implants which could be involved in the physiological recovery induced by these implants. For this purpose, we transected the MLF in adult rats and implanted NPCs at the lesion site. Changes induced by the lesion and/or the implant in the expression of VEGF, BDNF, NT-3 and NGF were analyzed in the axotomized population of abducens internuclear neurons. In addition, we also evaluated the expression of these neurotrophic molecules by the implanted NPCs. The findings of this study indicate that axotomy induces changes in the expression of neurotrophic factors by injured central neurons that can be differentially modulated by NPC implants. We propose that the neurotrophic regulation promoted by NPCs might contribute to the restorative effects of NPC implants.

## Materials and Methods

Experiments were performed in adult and 7 day postnatal (P7) Wistar rats in accordance with the guidelines of the European Union (86/609/EU) and Spanish law (R.D. 120/2005 BOE 252/34367-91, 2005) for the use and care of laboratory animals. Surgical procedures used in this study were approved by the ethics committee of Universidad de Sevilla.

### Neural Progenitor Cell Culture and Infection

**Figure 1:**
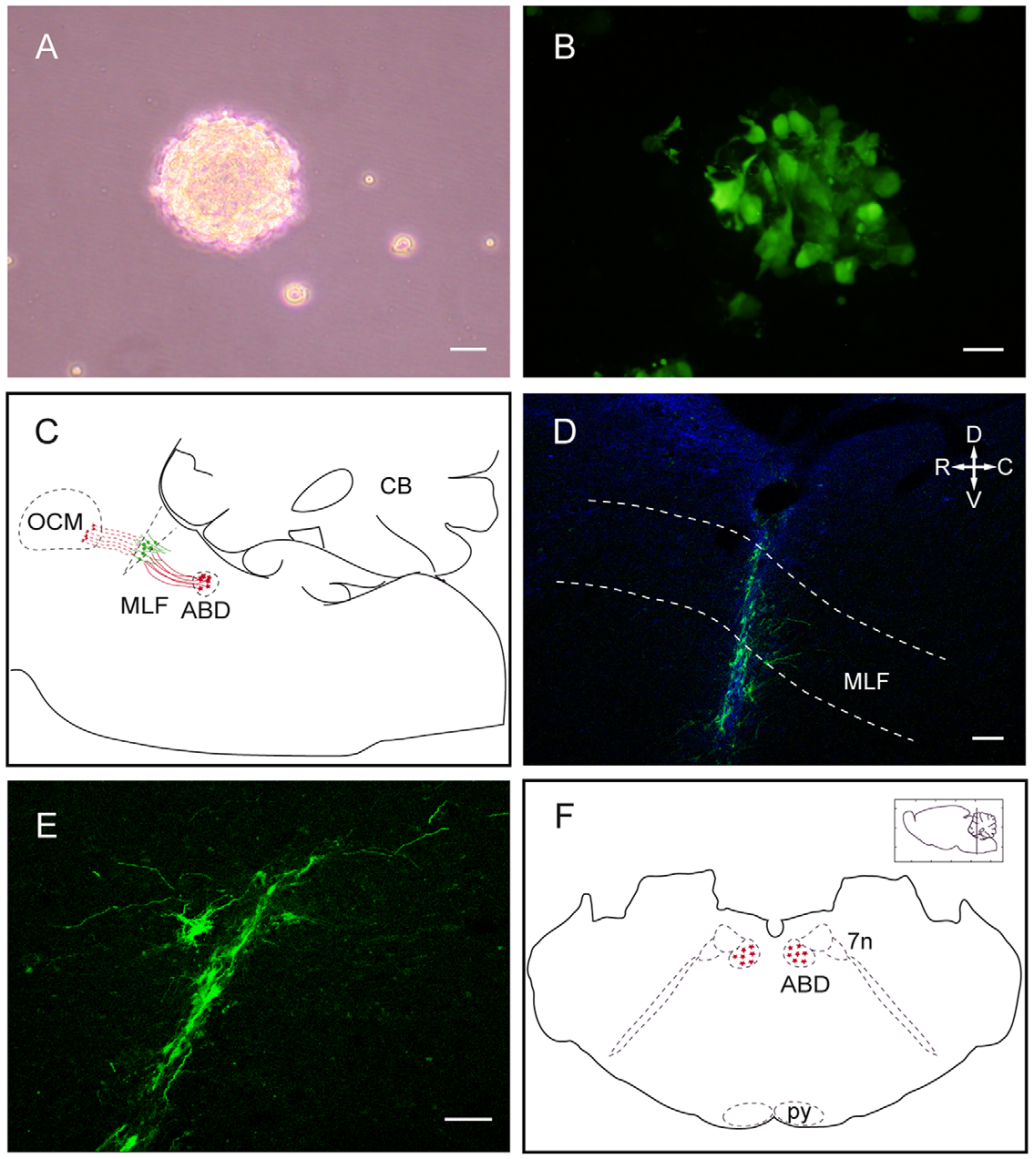
Neural progenitor cell culture, axotomy and cell implant. A. Floating neurosphere obtained from neural progenitors of the postnatal rat subventricular zone (SVZ). Scale bar: 25 µm. B. GFP-expressing cells (in green) in a SVZ-derived neurosphere. Scale bar: 25 µm. C. Schematic drawing of a rat parasagittal brainstem section showing the location of the medial longitudinal fascicle (MLF) transection and cell implant. Abducens internuclear neurons are represented in red and implanted neural progenitor cells in green. Axons of abducens internuclear neurons (red lines) course through the MLF towards the contralateral oculomotor nucleus. The distal stump of disrupted axons are represented in red dashed lines. D. Confocal microscopy image of a parasagittal brainstem section showing the implanted cells labeled with GFP (in green) at the site of axotomy. Dashed lines indicate the approximate dorso-ventral limits of the MLF. Scale bar: 100 µm. E. Higher magnification image of implanted GFP-labeled cells. Dorso-ventral and rostro-caudal orientation as in D. Scale bar: 50 µm. F. Schematic representation of a rat coronal section through the pons showing the abducens nucleus location. Abducens internuclear neuron somata are represented in red. **Abbreviations:** ABD: abducens nucleus; C: caudal; CB: cerebellum; D: dorsal; GFP: green fluorescent protein; MLF: medial longitudinal fascicle; OCM: oculomotor nucleus; py: pyramidal tract; R: rostral; V: ventral; 7n: facial nerve.

Neural progenitors were isolated from the SVZ of postnatal rats (P7) and were expanded in the form of neurospheres essentially as reported [31]. Briefly, the lateral walls of the lateral ventricles were removed and enzymatically dissociated with 1 mg/ml trypsin at 37°C for 15 min. The tissue was then centrifuged at 150 g for 5 min, rinsed in Dulbecco’s modified Eagle’s medium/F12 medium 1∶1 (DF-12) and centrifuged again in the same conditions. Then, the cells were resuspended in DF-12 containing 0.7 mg/ml ovomucoid, and mechanically disaggregated with a fire polished Pasteur pipette. The dissociated cells were centrifuged, resuspended in defined medium (DM: DF-12 containing B-27 supplement, 2 mM glutamine, 100 units/ml penicillin, 100 µg/ml streptomycin and 0.25 µg/ml amphotericin B) supplemented with 20 ng/ml EGF and 10 ng/ml FGF-2, and maintained in an atmosphere of 5% CO_2_, at 37°C. After 1–2 days, cell aggregates known as neurospheres were formed (Figure 1A). Cells were subcultured 48 hours after isolation and then every 3–4 days. Neurosphere-derived cells were immunopositive for the neural precursor marker nestin and were able to generate glial and neuronal cells when they were induced to differentiate after plating on adhesive substrate (supporting information S1 and Figure S1).

Neurospheres were infected with a lentiviral vector containing a reporter gene encoding for the green fluorescent protein (GFP) under the control of the cytomegalovirus (CMV) promoter (generously provided by Dr. David Macías, Hospital Universitario Virgen del Rocío, University of Seville; lentivirus titer: 3×10^6^ IFU/ml) with a multiplicity of infection of 3. Neurosphere-derived cells were incubated for 8 hours with virus-added DM in T25 flasks containing 250,000 cells. Virus-containing medium was then removed and replaced with fresh DM. After 48–72 hours of virus incubation, cells from neurospheres expressed GFP as confirmed by direct visualization in a Zeiss LSM 7 DUO confocal microscope (Figure 1B). The phenotype of the neurosphere-derived cells was not affected by the infection since they maintained the same ability to generate neurons and glial cells in differentiating conditions (data not shown).

### Transection of the Medial Longitudinal Fascicle and Neural Progenitor Cell Implantation

Under general anesthesia (4% chloral hydrate, 1 ml/100 g, i.p.), adult rats were placed on a stereotaxic frame and a window trephined in the occipital bone. The right MLF was transected by means of a 750-µm width microblade aimed with a 45° anterior angle at the following stereotaxic coordinates: 3.5 mm caudal to lambda and 0 mm lateral [32]. Figure 1C shows the site of axotomy in a schematic drawing of a rat brainstem parasagittal section. Then, lesioned rats were divided into two groups according to the type of procedure performed after the axotomy: the implanted group and the axotomized group. Rats of the implanted group received an injection of GFP-expressing neurosphere-derived cells (one µl of a suspension of 50,000 viable cells/µl prepared in DF-12). Cells were injected with a Hamilton microsyringe coupled to a glass capillary in the same coordinates of the lesion (Figures 1C–E). Rats from the axotomized group received only vehicle injection (one µl of DF-12) after the MLF transection.

The unilateral transection of the right MLF disrupts the axons of the internuclear neurons of the left abducens nucleus. As the left MLF was not transected, right side abducens internuclear neurons remained unlesioned and were used as control neurons throughout this study.

Eight weeks after lesion followed by cell implant or vehicle injection, animals were perfused transcardially under deep anesthesia (sodium pentobarbital, 50 mg/kg, i.p.) with 100 ml of physiological saline followed by 250 ml of 4% paraformaldehyde in 0.1 M sodium phosphate buffer, pH 7.4. The brainstem was removed and cryoprotected by immersion in a solution of 30% sucrose in sodium phosphate-buffered saline (PBS) until it sank. Then, for cryostat sectioning, the brainstem was cut in two pieces: the more rostral one, containing the site of lesion in the MLF, was sectioned sagitally (Figure 1C–E) and the caudal one, containing the abducens nucleus, was sectioned coronally (schematically represented in Figure 1F). All the sections were 40-µm thick. Sections were divided in parallel series to be processed for different immunostainings.

### Immunohistochemistry

After washing in PBS, coronal sections containing the abducens nucleus were incubated with 1% sodium borohydride for 10 min for antigen retrieval. They were rinsed again in PBS and incubated for 1 hour in a blocking solution consisting of 5% normal donkey serum in PBS with 0.01% Triton X-100 (PBS-T). Following the blocking treatment, sections were incubated overnight at room temperature with one of the following primary antibodies: rabbit anti-VEGF (Santa Cruz Biotechnology, sc-507, 1∶100), rabbit anti-BDNF (Santa Cruz Biotechnology, sc-546, 1∶400) or rabbit anti-NT-3 (Santa Cruz Biotechnology, sc-547, 1∶400), prepared in blocking solution. After several rinses in PBS, sections were incubated with a biotinylated secondary antibody in PBS-T (biotinylated anti-rabbit IgG Jackson ImmunoResearch, 1∶500), washed again and finally transferred for 45 minutes to a solution containing streptavidin-FITC (Jackson ImmunoResearch, 1∶800). Then, in order to identify abducens internuclear neurons, subsequent immunohistochemistry for the detection of calretinin was performed since this protein is selectively expressed within the abducens nucleus by this population of neurons [33]. The primary antibody was a goat anti-calretinin from Swant (1∶500) and the antibody binding was made visible by incubation with an anti-goat-TRITC (Jackson ImmunoResearch, 1∶100). In other experiments, when the detection of abducens motoneurons was required, a goat anti-choline acetyltransferase (ChAT) antibody (Millipore, 1∶500) followed by anti-goat-TRITC incubation was used. Sections were then washed for several times, mounted on glass slides and coverslipped with a 0.1 M solution of propyl gallate prepared in glycerol:PBS (9∶1).

The immunohistochemical protocol for NGF varied slightly since a different antigen retrieval method was needed, which consisted of incubating the sections in 0.01 M sodium citrate buffer, pH 6, at 74°C for 40 min. The remaining protocol was the same as described above except that incubation with the primary antibody (rabbit anti-NGF; Santa Cruz Biotechnology, sc- 548, 1∶100) lasted for 72 hours at 4°C. Finally, calretinin immunohistochemistry and mounting on slides were performed as described above.

It is important to point out that, in order to avoid differences in immunostaining intensity due to methodological procedures, series of sections from implanted and axotomized groups were always processed simultaneously for each neurotrophic factor.

To analyze neurotrophic factor expression by implanted cells, parasagittal sections containing the lesion and implant site were processed for single immunohistochemistry for VEGF, BDNF, NT-3 or NGF, following the same described procedure but a streptavidin-DyLight649 was used (Jackson ImmunoResearch, 1∶800) instead of a FITC-coupled streptavidin, since implanted cells were already labeled in green by their GFP expression.

### Antibody Characterization

For positive controls, the immunohistochemistry protocol was performed in brain sections that contained areas previously reported as immunopositive for each of the neurotrophic factors used in this study.

Negative controls carried out by omission of the primary antibodies resulted in absence of staining in all cases. Additional negative controls were performed by preadsorption of the antibodies with their blocking peptides. Specifically, each antibody and its corresponding immunizing peptide were mixed for 2 hours at a 1∶20 proportion before being used for the immunohistochemical protocol. Sections incubated with this preadsorbed solution showed no specific staining.

For the VEGF primary antibody, a blocking peptide was not available by the provider. Then, as negative control, the primary antibody incubation was replaced by incubation with normal rabbit IgG (Jackson ImmunoResearch) at the same concentration than the primary antibody. Again, no specific labeling was found.

### Confocal Microscopy and Optical Density Quantification

Confocal microscopy images of abducens neurons were captured in order to analyze differences between groups in the intensity of the VEGF, BDNF, NT-3 and NGF immunostainings.

For each neurotrophic factor, all abducens neurons identified in one out of three coronal sections from each animal were captured with a confocal laser scanning microscope (Zeiss LSM 7 DUO) at 63X magnification and 8-bit resolution. Different focal planes containing the cell nucleus and separated 2 µm in the z axis were scanned by exciting the FITC and TRITC fluorophores with 488-nm argon and DPSS 561-nm lasers, respectively, with a pinhole aperture of 1 Airy Unit. Before scanning, gray scales were adjusted to maximize their dynamic range. Acquisition settings were kept constant during the image capture of neurons from the left (lesioned) and right (unlesioned) abducens nucleus of the same section.

Captured images were analyzed with ImageJ (NIH). In order to determine the intensity of the immunostaining, we calculated the mean gray value of the cell immunolabeling (i.e., the optical density) by manually outlining the cell cytoplasm in the corresponding scanned images. Optical density measurements have been demonstrated to correlate significantly with the amount of protein present in the brain tissue [34] and have been widely used by other authors to compare protein levels between groups [35], [36], [37], [38]. Optical density values for VEGF, BDNF, NT-3 and NGF immunoreactivies were determined for neurons of the lesioned and the unlesioned (control) sides of both axotomized and implanted animals and data were finally expressed as percentages relative to the control side.

### Statistics

Comparisons between groups were carried out in Sigma Plot 11 (Systat Software) by using one-way ANOVA followed by post-hoc multiple comparisons (Dunńs method) at a significant level of p<0.05.

## Results

### Antibody Characterization

Positive and negative controls were carried out to test the specificity of the antibodies used in this study for the immunoidentification of the neurotrophic factors BDNF, NT-3, NGF and VEGF.

**Figure 2:**
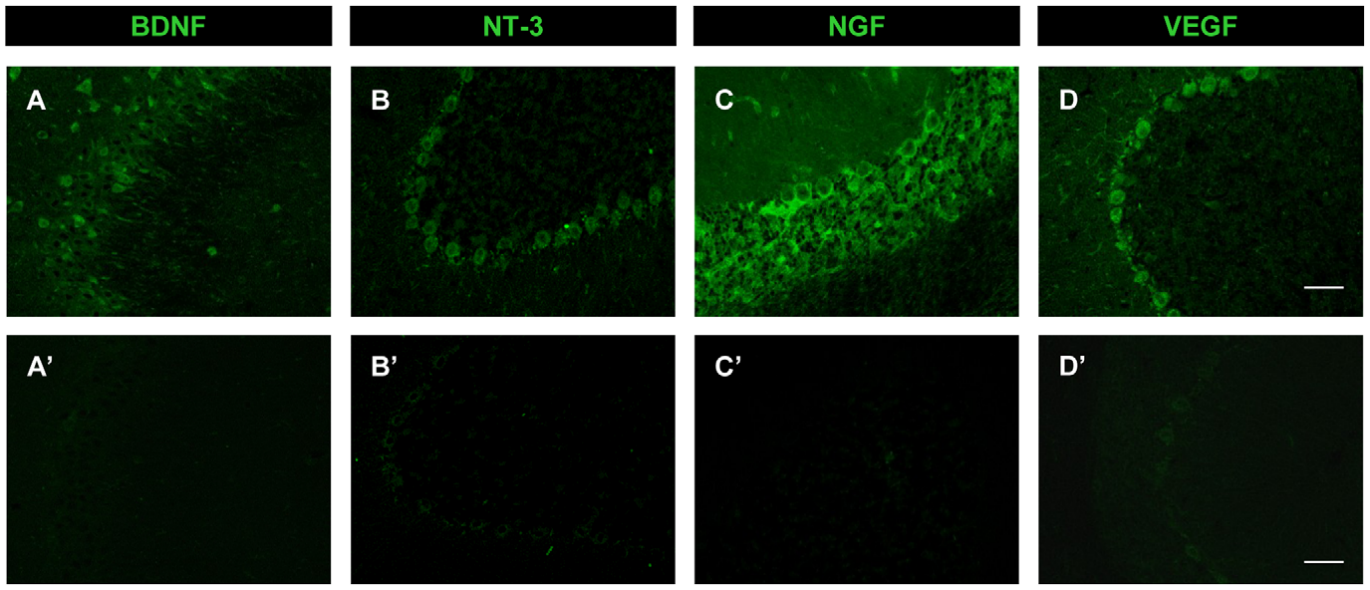
Antibody characterization. Confocal microscopy images of rat coronal sections through the hippocampus (A, A’) and cerebellum (B, B’, C, C’) showing immunoreactivity to BDNF, NT-3, and NGF in positive controls (A, B and C, respectively) and in their respective blocking peptide-preadsorbed negative controls (A’, B’ and C’). D and D’. Confocal microscopy images of cerebellar coronal sections showing VEGF immunoreactivity in a positive control (D) and a negative control consisting of tissue pre-incubation with normal rabbit IgG instead of VEGF antibody (D’). Scale bars: 50 µm.

Initially, the immunohistochemistry protocol and the antibody titration were established in sections containing brain regions in which the expression of each factor has been previously described in the literature [30], [39], [40], [41], [42], [43]. Thus, immunoreactivity for BDNF was found in rat hippocampus and the immunostaining was not evident when the antibody was preadsorbed with its immunizing peptide (Figures 2A and 2A’). Positive results were obtained for NT-3 immunostaining in cerebellar Purkinje cells (Figure 2B) and in hippocampus (not shown), and specific staining was absent when sections were incubated with the preadsorbed antibody (Figure 2B’). NGF immunodetection required a more intense antigen retrieval method after which its expression was evident in cerebellum (Figure 2C) and in hippocampus (not shown). Again, preincubation with the blocking peptide led to negative staining (Figure 2C’).

VEGF expression was conspicuous in Purkinje cells of the cerebellum (Figure 2D) and in many other brain regions such as cortex or hippocampus (not shown). As the VEGF blocking peptide was not available (see Materials and Methods), sections were preincubated with normal rabbit IgG instead of the rabbit-made primary antibody, which also produced negative labeling in the cerebellum (Figure 2D’) and in the other described regions.

### VEGF Immunoreactivity is Increased in Lesioned Abducens Internuclear Neurons of Implanted Animals

**Figure 3:**
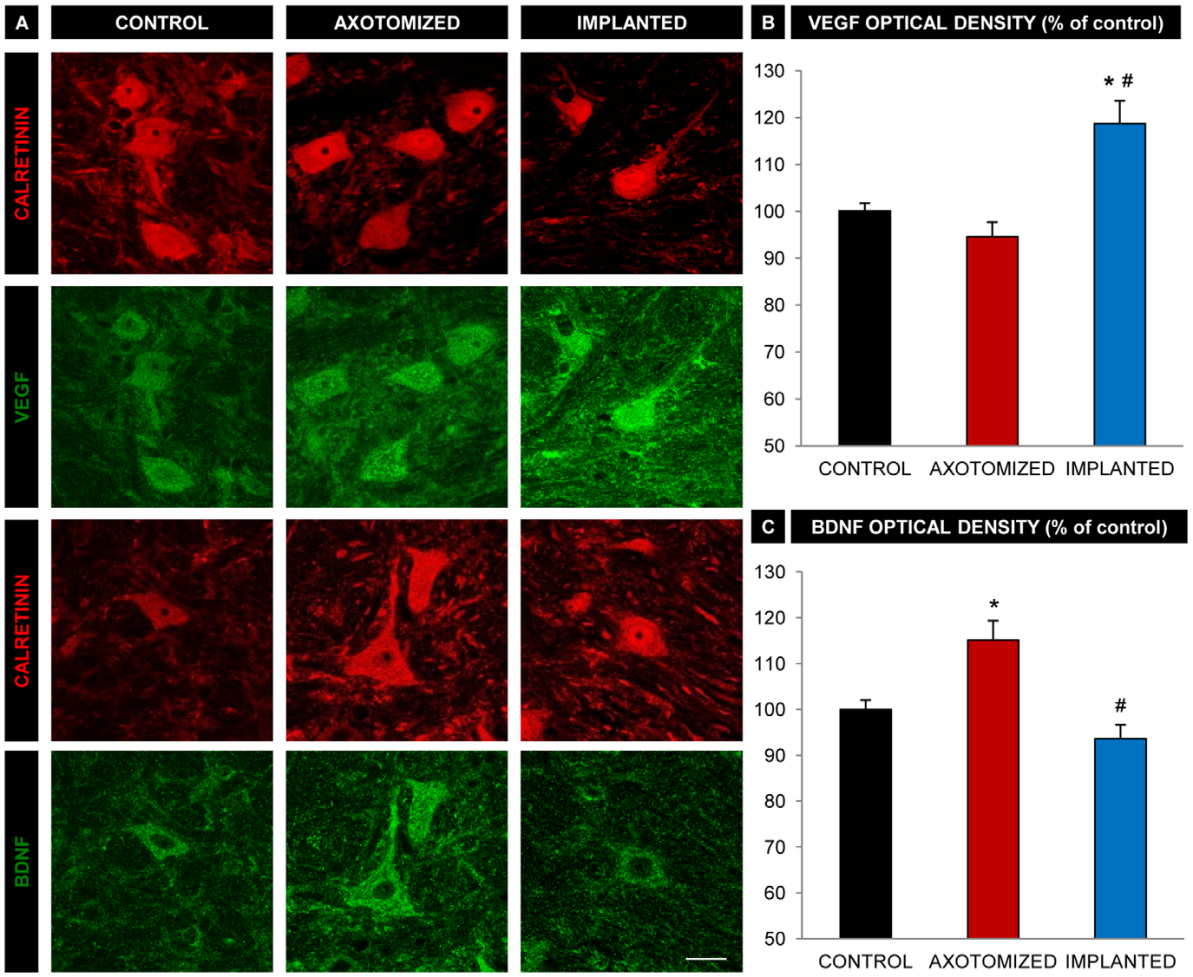
VEGF and BDNF immunoreactivity in abducens interneurons. A. Confocal microscopy images of abducens internuclear neurons (immunopositive for calretinin, in red) showing double immunoreactivity against either VEGF (in green, second row of images) or BDNF (in green, fourth row). Examples of neurons from the different experimental conditions are shown: non lesioned neurons (CONTROL column), lesioned neurons from the axotomized group (AXOTOMIZED column) and lesioned neurons from the implanted group (IMPLANTED column). Bar: 20 µm. B. Optical density quantification of VEGF immunoreactivity in unlesioned neurons (CONTROL), lesioned neurons from the axotomized group (AXOTOMIZED) and lesioned neurons from the implanted group (IMPLANTED). Values are expressed as percentages relative to control (unlesioned neurons). Bars represent the mean ± SEM of 60–67 neurons analyzed from five different animals in each group. * and # indicate significant differences with respect to the control and the axotomized group, respectively (ANOVA test followed by Dunn’s method for multiple pairwise comparisons, p<0.05). C. Same as B, but for BDNF immunoreactivity. Bars represent the mean ± SEM of 32–54 neurons analyzed from four different animals in each group. * and # indicate significant differences with respect to the control and the axotomized group, respectively (ANOVA test followed by Dunn’s method for multiple pairwise comparisons, p<0.05).

As previously described, calretinin constitutes a good marker of the internuclear neurons located in the abducens nucleus [33]. Therefore, we used calretinin immunolabeling for the identification of this cell type. In all experimental groups (i.e., control, axotomized and implanted), the double immunofluorescence performed against calretinin and VEGF revealed that virtually all calretinin-positive abducens cells were also strongly immunoreactive for VEGF (Figure 3A). We further evaluated the intensity of the immunolabeling by quantifying the optical density within the neuronal soma of abducens internuclear neurons, as described in Materials and Methods. Thus, axotomy did not affect the intensity of VEGF immunoreaction, since injured neurons showed similar values of VEGF optical density in comparison with control cells (Figure 3A and 3B). Interestingly, abducens internuclear neurons from animals that received the NPC implants showed a significant increase in VEGF optical density, as compared with both control and axotomized cells (Figure 3B; p<0.05, ANOVA test; n = 60–67 neurons in each group obtained from 5 animals).

### BDNF Immunoreactivity is Increased in Lesioned Neurons of Axotomized Animals but not of Implanted Animals

All calretinin-immunopositive neurons examined in the abducens nucleus also showed a strong immunoreactivity against BDNF, as observed in the three experimental groups (Figure 3A). However, the intensity of BDNF immunolabeling in abducens internuclear neurons changed eight weeks after MLF transection. Thus, BDNF optical density raised significantly in axotomized neurons in relation to control values (Figure 3C). In contrast, axotomized neurons from animals provided with NPC implants did not exhibit any change in BDNF optical density, as compared with the control group (Figure 3C; n = 32–54 neurons analyzed in each group from 4 animals).

### Changes in VEGF and BDNF Immunoreactivity in the Abducens Nucleus are Selective for the Injured Cell Population

**Figure 4:**
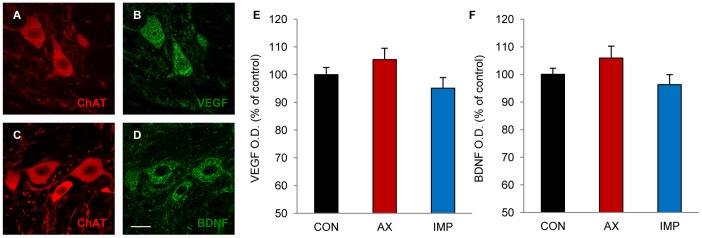
VEGF and BDNF immunoreactivity in abducens motoneurons. A to D. Confocal microscopy images of control abducens motoneurons showing double immunolabeling for ChAT (A, in red) and VEGF (B, in green), or for ChAT (C, in red) and BDNF (D, in green). Scale bar: 20 µm. E and F. Optical density (O.D.) quantification of VEGF immunoreactivity (E) and BDNF immunoreactivity (F) in abducens motoneurons from either the unlesioned side (CON), the lesioned side of the axotomized group (AX) or the lesioned side of the implanted group (IMP). Values represent percentages with respect to control (motoneurons from the unlesioned side). Bars show the mean ± SEM of 58–79 motoneurons analyzed from three different animals in each group. No significant differences were obtained between groups (ANOVA test followed by Dunn’s method for multiple pairwise comparisons).

In order to test whether the effects of central axotomy and NPC implants on VEGF and BDNF expression were restricted to the injured neuronal population within the abducens nucleus (i.e., the abducens internuclear neurons) we also examined VEGF and BDNF immunoreactivity in the motoneurons of the same nucleus, whose axons were left intact. Abducens motoneurons were identified by their ChAT immunostaining (Figure 4A and C). Both VEGF and BDNF were expressed by virtually all abducens motoneurons analyzed in the three experimental groups (n = 58–79 motoneurons per group obtained from 3 animals) (Figure 4A–D), but their optical density values were not affected by either the MLF transection or the cell implantation (Figure 4E and 4F). Therefore, abducens motoneurons did not modify their VEGF or BDNF expression in response to the transected fascicle and NPC implants, so that the changes found in the immunoreactivity for these two neurotrophic molecules were specific to the population of injured abducens internuclear neurons.

### Implanted Animals do not show Changes in NT-3 or NGF Immunoreactivity with Respect to Axotomized Animals

**Figure 5:**
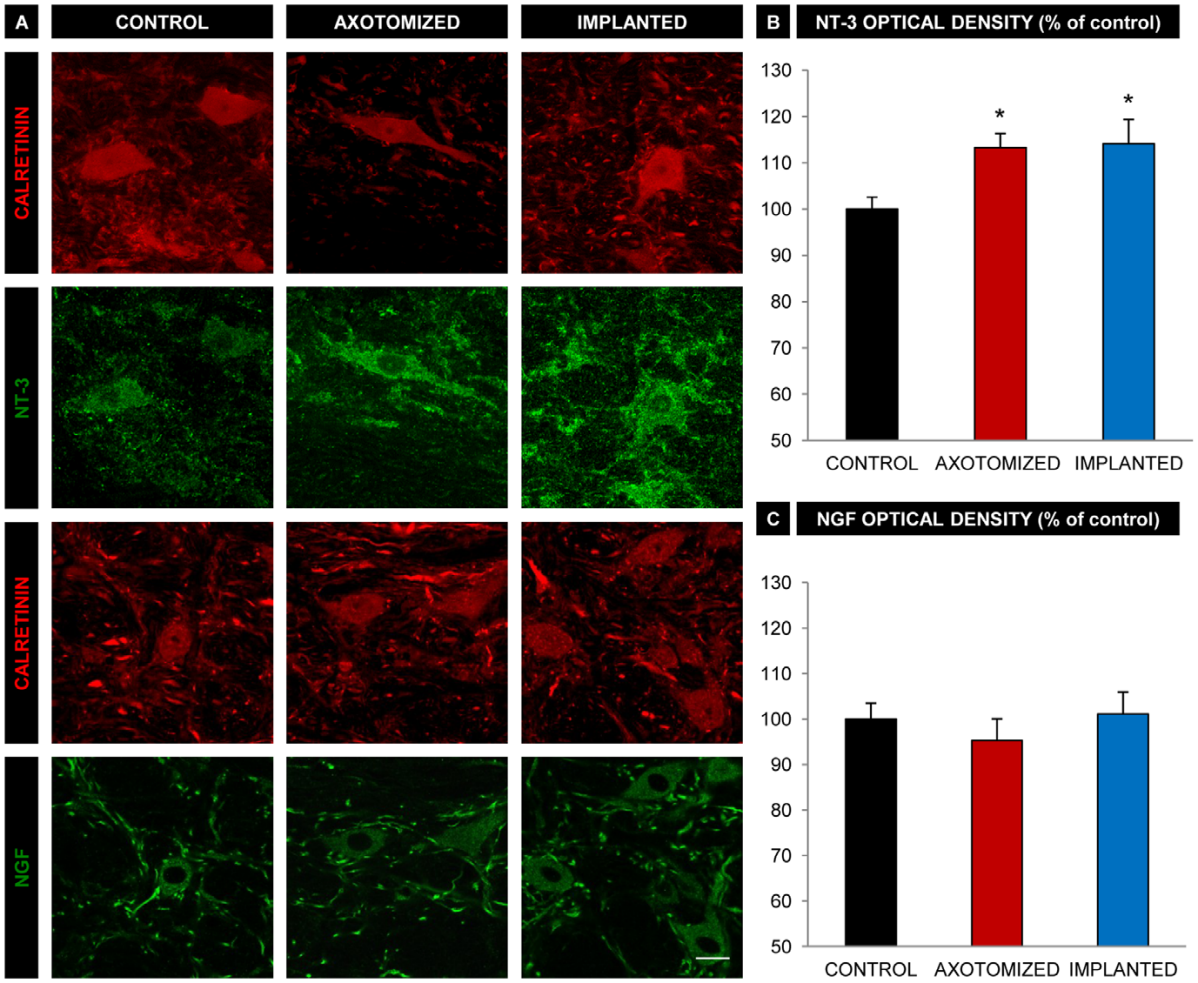
NT-3 and NGF immunoreactivity in abducens interneurons. A. Confocal images of abducens interneurons (immunopositive for calretinin, in red) showing double immunoreactivity against either NT-3 (in green, second row of images) or NGF (in green, fourth row). Examples of neurons from the different experimental conditions are shown: non lesioned neurons (CONTROL column), lesioned neurons from the axotomized group (AXOTOMIZED column) and lesioned neurons from the implanted group (IMPLANTED column). Scale bar: 20 µm B. Optical density quantification of NT-3 immunoreactivity in unlesioned neurons (CONTROL), lesioned neurons from the axotomized group (AXOTOMIZED) and lesioned neurons from the implanted group (IMPLANTED). Values are expressed as percentages with respect to control (unlesioned neurons). Bars represent the mean ± SEM of 25–38 neurons analyzed from four different animals in each group. *, p<0.05 compared to the control group, no significant differences were obtained between the axotomized and the implanted groups (ANOVA test followed by Dunn’s method for multiple pairwise comparisons, p<0.05). C. Same as B, but for NGF immunoreactivity. Bars represent the mean ± SEM of 26–54 neurons analyzed from three different animals in each group. Note that there were no significant differences between groups (ANOVA test followed by Dunn’s method for multiple pairwise comparisons, p<0.05).

Abducens internuclear neurons showed a weak immunostaining against NT-3 (Figure 5A). The analysis of optical density after NT-3 immunolabeling revealed a significant increase of this parameter in the cell body of abducens internuclear neurons as a result of their axotomy (Figure 5B). Abducens internuclear neurons axotomized but provided with the NPC implants also showed a significant increase in NT-3 optical density as compared with control, which was similar to that exhibited by the axotomized group (Figure 5B). The number of calretinin-immunopositive neurons that appeared doubly labeled following the NT-3 immunofluorescence was similar in the three experimental groups and close to 100% (n = 25–38 neurons in each group obtained from 4 animals).

NGF immunoreactivity was mainly confined to the neuropil of the abducens nucleus, showing faintly-stained cell bodies (Figure 5A). Optical density quantification evidenced no significant change between groups in the intensity of NGF immunoreactivity, so that axotomized abducens internuclear neurons with or without the NPC implant showed values of NGF optical density that were similar to control (Figure 5C; n = 26–54 neurons per group obtained from 3 animals).

### VEGF Expression by the Implanted Neural Progenitor Cells

**Figure 6:**
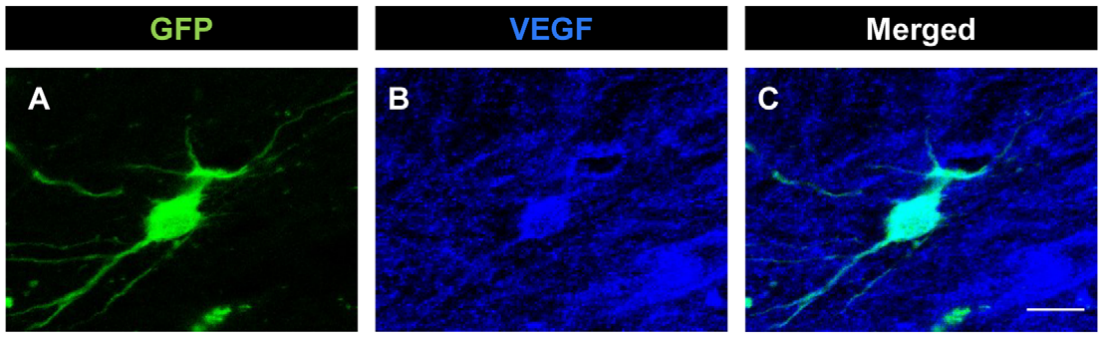
VEGF immunoreactivity in neural progenitor implanted cells. Confocal microscopy images of an implanted cell at the lesion site identified by its GFP expression (A, in green), which was also immunopositive for VEGF (B, in blue). C shows the merged image. Scale bar: 10 µm.

We also aimed to explore whether implanted cells in the lesion site might constitute a retrograde source of neurotrophic factors for the axotomized abducens internuclear neurons, due to the close proximity between the proximal stump of sectioned axons and the implanted NPCs. For that, we performed VEGF, BDNF, NT-3 and NGF immunohistochemistry in brainstem parasagittal sections containing the lesion and NPC implantation site (Figure 1C–E). Implanted NPCs were identified by its GFP labeling. Approximately 25% of the GFP-positive NPCs examined appeared also labeled following VEGF immunoreactivity. Figure 6 illustrates an example of a NPC implanted at the lesion site which showed double labeling for both GFP and VEGF immunofluorescence. In contrast, none of the analyzed GFP-positive cells in the implant was found to be immunopositive for BDNF, NT-3 or NGF. Therefore, among all neurotrophic factors examined, VEGF was the only that could be delivered retrogradely from implanted cells to axotomized neurons.

## Discussion

In this study, we have demonstrated that VEGF and BDNF expression in axotomized neurons can be modulated differentially by NPCs implanted in the site of injury. In addition, NPCs derived from the SVZ are able to express VEGF after implantation in the damaged brain. Based on the recently-demonstrated and relevant functions of VEGF in neuroprotection and synaptic modulation, we propose that this neurotrophic factor could play an important role in the benefitial effects of NPC implants on axotomized abducens internuclear neurons.

Transection of the MLF leaves abducens internuclear neurons disconnected from their natural target in the oculomotor nucleus. The axotomized neurons exhibit a profound removal of inhibitory and excitatory synapses and a reduction in eye position and velocity sensitivities [2], [11]. In a previous work, we have shown that NPCs implanted in the cat after transection of the MLF induce a noticeable recovery in synaptic coverage and firing properties altered by the axotomy [19]. As described in other lesion models, NPCs might induce changes of protein expression in lesioned cells which could be determinant in the mechanism of NPC-induced recovery [21], [22], [23]. Therefore, we questioned whether NPC implants were able to modify neurotrophic factor expression in axotomized abducens internuclear neurons by means of immunohistochemical tools. First, we had to establish reliable immunohistochemical protocols with specific antibodies raised against VEGF, BDNF, NT-3 and NGF. Positive controls revealed immunolabeling in brain regions previously described to be immunopositive for the mentioned factors such as hippocampus, cerebral cortex or cerebellum [30], [39], [40], [41], [42], [43]. Negative controls were performed by preadsorption of the antibody with the blocking peptide (for BDNF, NT-3 or NGF) or with normal IgG in substitution of the antibody (for VEGF). It is noteworthy that we never obtained specific labeling in these cases which indicated that antibodies used for this study were reliable and specific.

In the control situation, we observed that all analyzed abducens internuclear neurons displayed an intense immunoreactivity for VEGF and BDNF, whereas NT-3 and NGF revealed a faint staining. However, this was not the case for other brainstem neurons which showed an intense immunoreactivity for NT-3 and NGF (data not illustrated). Therefore, the intense labeling found in abducens internuclear neurons specifically after VEGF and BDNF immunoreactivity suggests that these two neurotrophic factors likely act as important molecules for the functional maintenance of this neuronal population, probably through autocrine or paracrine pathways, although the retrograde and/or anterograde routes can not be discarded.

After axotomy, abducens internuclear neurons did not modify their immunoreactivity to VEGF. However, VEGF content was significantly increased in these neurons when NPCs were implanted after axotomy. Two possible explanations may account for this finding: i) VEGF might have been retrogradely transported by the sectioned axons and accumulated in the soma, and ii) damaged neurons might have increased their VEGF expression in implanted animals. Since some of the implanted cells were VEGF-immunoreactive and abducens internuclear neurons express the receptor for VEGF, Flk-1 (unpublished data), the first possibility is feasible. With regard to the second possibility, it is well established that the new microenvironment created by the cross-talk between implanted and host tissue cells can induce changes in the neuronal expression of certain molecules [21], [22], [23], [44]. Therefore, it is also possible that an increase in VEGF expression was induced in axotomized cells as a result of molecular interactions derived from this new microenvironment. Independently from the source, damaged neurons of implanted animals might benefit from the VEGF increased content. Indeed, recent evidence relates increased VEGF expression with neuroprotective actions. For instance, injured neurons after insults such as ischemia or brain seizures increase their VEGF expression and probably this might operate as an endogenous protective mechanism that reduces neuronal excitability [27], [29]. Also, overexpression of VEGF is known to delay motor neuron degeneration in animal models of amyotrophic lateral sclerosis [28], [45]. In the cat, NPC implants are able to restore most of the axotomy-induced alterations in firing properties and synaptic inputs of abducens internuclear neurons [19]. We propose that VEGF could be involved in the neuroprotective actions exerted by NPC implants in axotomized animals although further experiments are needed in order to elucidate this issue.

Abducens internuclear neurons of the axotomized group exhibited a significant increase in BDNF and NT-3 content eight weeks after MLF transection. We have previously demonstrated that abducens internuclear neurons express the trk receptors for BDNF and NT-3 (trkB and trkC, respectively) [15]. Therefore, by increasing BDNF and NT-3 expression, axotomized neurons might compensate for the lack of target-derived trophic support via autocrine or paracrine pathways [7], [8], [46]. However, in the implanted group, BDNF content of abducens internuclear neurons was not increased after axotomy and remained in values similar to healthy neurons. Probably, other endogenously-released molecules in the new microenvironment generated by the NPC implant might replace BDNF-dependent neurotrophic actions, and consequently this neurotrophin might be down-regulated as compared to the axotomy situation. This was not the case for NT-3, since this neurotrophin increased significantly in both the axotomized and implanted groups and, therefore, neither the NPCs nor other cells from the host tissue that interacted with the NPCs could modify the axotomy-induced increase in NT-3 content. Since NGF immunoreactivity in abducens internuclear neurons was low and, in addition, was not affected by either the axotomy or the NPC implants, it seems that this neurotrophin is much less relevant in control as well as after lesion and implant.

A noticeably finding of the present study was that the motoneurons of the abducens nucleus, which lie intermingled with the internuclear neurons, did not show any change in the intensity of their immunostaining for either VEGF or BDNF. Since the same methodological protocol was used for both cell types, these data clearly validate the modifications found in the population of abducens internuclear neurons after NPC implants and, moreover, demonstrate that changes in neurotrophic factors were exclusive for those neurons that were axotomized by the lesion procedure.

As stated before, implanted cells might constitute a new source of trophic factors for axotomized cells. In fact, NPCs from the SVZ express VEGF in the neurogenic niche [24] and also when they are cultured in the form of neurospheres [47]. Fagerlund et al. [18] have recently reported that NPCs derived from the SVZ and transplanted in the lesioned hypoglossal nucleus express VEGF and promote motor neuron survival. In line with those findings, we pursued to investigate in our CNS lesion model whether SVZ neurosphere-derived cells were able to express VEGF or other neurotrophic factors eight weeks after implantation in the host lesioned tissue. Our results showed that none of the analyzed implanted cells were immunoreactive for BDNF, NT-3 or NGF, but a moderate percentage (approximately 25%) was immunopositive for VEGF, which indicated that implanted NPCs could release VEGF in the proximity of the proximal stumps of severed axons.

In summary, this study demonstrates that NPCs implanted in the injured brain express VEGF eight weeks after implantation in the host lesioned tissue and are able to modulate VEGF and BDNF expression in lesioned neurons. We propose that the increased VEGF expression promoted by NPCs in injured neurons might be involved in the restorative effects of NPC implants.

## Supporting Information

Figure S1 Characterization of neurosphere cultures. A-B. Phase-contrast microscopy images of floating neurospheres (A) and dissociated neurosphere cells grown as a monolayer on a poly-lysine substrate (B). C, E, G. Fluorescence microscopy images of neurospheres immunostained with antibodies against nestin (C, red), βIII-tubulin (E, arrowhead, red), GFAP (E, arrow, green) and NG-2 (G, green) and counterstained with DAPI (blue). D, F, H. Fluorescence microscopy images of adhered cells immunostained with antibodies against nestin (D, red), βIII-tubulin (F, arrowhead, red), GFAP (F, arrow, green) and NG-2 (H, green) and counterstained with DAPI (blue). Scale bars: 50 µm in A, B and D; 25 µm in C and F; 15 µm in E, G and H.
(TIF)

Supporting Information S1 Characterization of neurosphere cultures.
(DOCX)
