# Supplementary figures and images for: Neural Progenitor Cell Implants Modulate Vascular Endothelial Growth Factor and Brain-Derived Neurotrophic Factor Expression in Rat Axotomized Neurons
Source: PLoS One. 2013 Jan 18;8(1):e54519. doi: 10.1371/journal.pone.0054519 (PMC3548797; doi:10.1371/journal.pone.0054519)

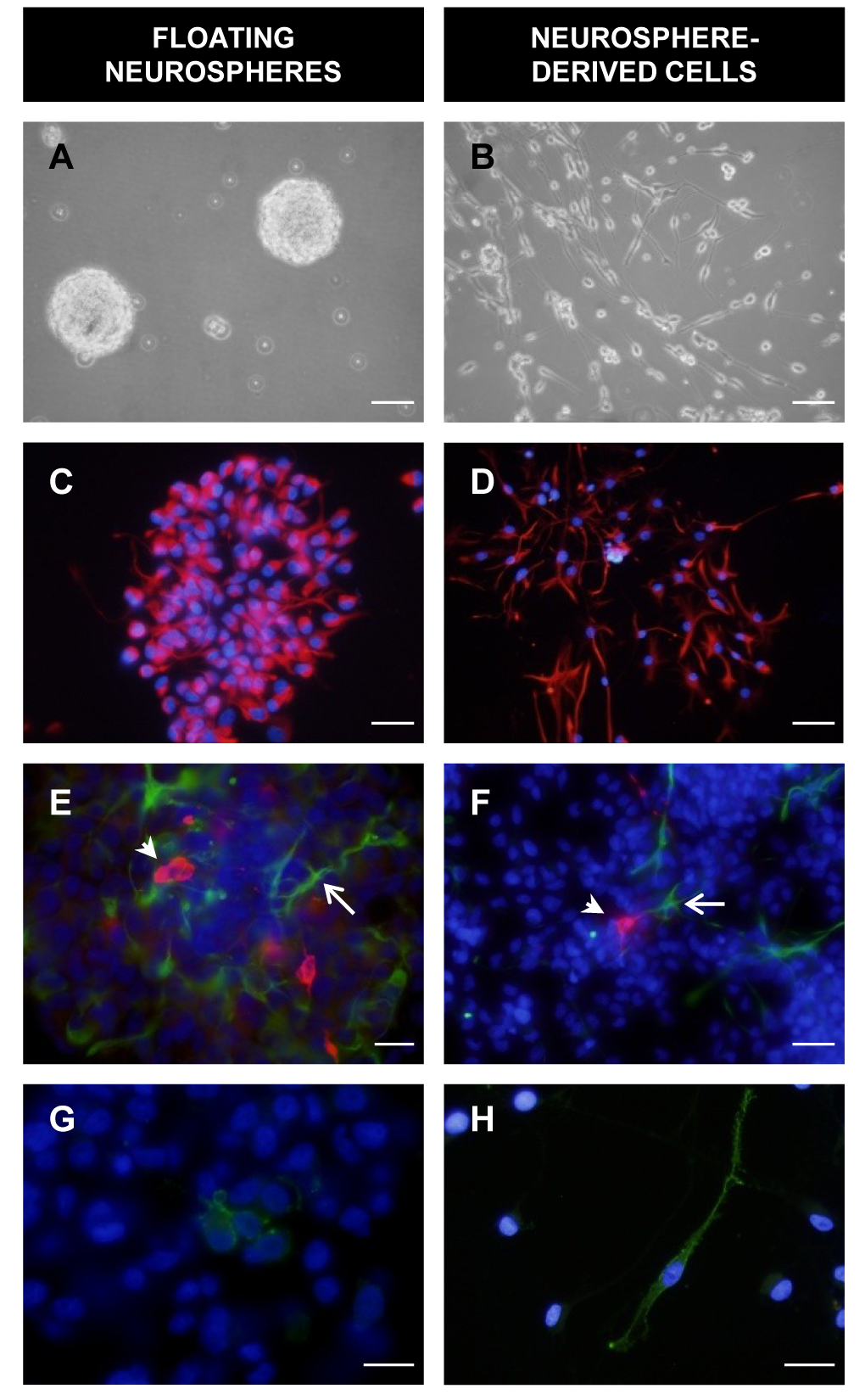

Supplement: Figure S1 — Characterization of neurosphere cultures. A-B. Phase-contrast microscopy images of floating neurospheres (A) and dissociated neurosphere cells grown as a monolayer on a poly-lysine substrate (B). C, E, G. Fluorescence microscopy images of neurospheres immunostained with antibodies against nestin (C, red), βIII-tubulin (E, arrowhead, red), GFAP (E, arrow, green) and NG-2 (G, green) and counterstained with DAPI (blue). D, F, H. Fluorescence microscopy images of adhered cells immunostained with antibodies against nestin (D, red), βIII-tubulin (F, arrowhead, red), GFAP (F, arrow, green) and NG-2 (H, green) and counterstained with DAPI (blue). Scale bars: 50 µm in A, B and D; 25 µm in C and F; 15 µm in E, G and H. (TIF) [file pone.0054519.s001.tif]
