# Supplementary material for: Neural Progenitor Cell Implants Modulate Vascular Endothelial Growth Factor and Brain-Derived Neurotrophic Factor Expression in Rat Axotomized Neurons
Source: PLoS One. 2013 Jan 18;8(1):e54519. doi: 10.1371/journal.pone.0054519 (PMC3548797; doi:10.1371/journal.pone.0054519)
Supplement: Supporting Information S1 — Characterization of neurosphere cultures. (DOCX) [file pone.0054519.s002.docx]

**Supporting Information S1**

**Characterization of neurosphere cultures**

To analyze differentiation, neurospheres (Figure S1A) were mechanically dissociated and seeded on poly-lysine-treated coverslips in DF-12 with 1% fetal calf serum for 4 hours to facilitate adhesion. Then, cultures were washed and maintained in DM supplemented with 20 ng/ml EGF and 10 ng/ml FGF-2 for 48 hours (Figure S1B). In other experiments, neurospheres were not mechanically dissociated but were placed directly on poly-lysine treated coverslips and left for only one hour in the incubator to facilitate adhesion. This will be referred to as floating neurospheres.

Both neurosphere-derived adhered cells and floating neurospheres were fixed with 4% paraformaldehyde in 0.1M phosphate buffer. Then, they were incubated for 30 min in a blocking solution containing 2.5% bovine serum albumin in phosphate-buffered saline and then in the primary (2 hours at room temperature) and, after rinsing, in the secondary (30 min at room temperature) antibodies prepared in blocking solution. After washing, cells were counterstained with 4’-6’-diamidino-2-phenylindole (DAPI, 0.1 µg/ml) for 10 min, washed again and mounted on slides with Vectashield. The primary antibodies used were: glial fibrillary acidic protein (GFAP, rabbit polyclonal, from DAKO, 1:500), β-III tubulin (mouse monoclonal, from Promega, 1:500), nestin (goat polyclonal, from Santa Cruz Biotechnology, 1:50) and NG-2 chondroitin sulfate proteoglycan (rabbit polyclonal, from Chemicon, 1:500). The secondary antibodies used were: anti-rabbit IgG labeled with FITC, anti-mouse IgG labeled with TRITC and anti-goat IgG labeled with FITC or with TRITC (all from Jackson ImmunoResearch, 1:100). Fluorescent signals were detected and analyzed with a BX61 Olympus epifluorescence microscope. Omission of primary antibodies resulted in the absence of detectable staining in all cases.

Floating neurospheres were constituted by nestin-positive precursor cells (Figure S1C) and most of them also contained a small number of daughter cells that expressed either astroglial (GFAP), early neuronal (β-III tubulin) or oligodendroglial precursor (NG-2) markers (Figures S1E and G) indicating that the neurosphere-forming cells were multipotent. Neurosphere-derived adhered cells also expressed all the mentioned phenotypes (Figures S1D, F and H).
